# Testing Human Sperm Chemotaxis: How to Detect Biased Motion in Population Assays

**DOI:** 10.1371/journal.pone.0032909

**Published:** 2012-03-08

**Authors:** Leah Armon, S. Roy Caplan, Michael Eisenbach, Benjamin M. Friedrich

**Affiliations:** 1 Department of Biological Chemistry, Weizmann Institute of Science, Rehovot, Israel; 2 Department of Materials and Interfaces, Weizmann Institute of Science, Rehovot, Israel; 3 Max Planck Institute for the Physics of Complex Systems, Dresden, Germany; Universita' del Piemonte Orientale, Italy

## Abstract

Biased motion of motile cells in a concentration gradient of a chemoattractant is frequently studied on the population level. This approach has been particularly employed in human sperm chemotactic assays, where the fraction of responsive cells is low and detection of biased motion depends on subtle differences. In these assays, statistical measures such as population odds ratios of swimming directions can be employed to infer chemotactic performance. Here, we report on an improved method to assess statistical significance of experimentally determined odds ratios and discuss the strong impact of data correlations that arise from the directional persistence of sperm swimming.

## Introduction

Chemotaxis refers to biased motion of cells or organisms in a concentration gradient of an external chemical factor, a so-called chemoattractant. Chemotaxis requires a fine-tuned interplay of motility and sensation and different navigation strategies are implemented by nature: bacterial chemotaxis relies on biased random walks for which the frequency of random tumbling events is regulated in response to temporal changes of chemoattractant concentration, which are perceived during swimming in a spatial concentration gradient [1]. Large and slowly moving cells like the slime mold Dictyostelium can detect a concentration gradient by spatial comparison along their cell length [2]. Finally, sperm cells from marine invertebrates with external fertilization use a clever strategy of temporal sampling a concentration field along circular and helical paths to enhance fertilization rates [3], [4]. Chemotaxis has also been demonstrated in human sperm [5], [6]. Human sperm chemotaxis also seems to rely on temporal sampling [7], but the precise navigation strategy remains elusive. Research on human sperm chemotaxis is further complicated by the fact that only a fraction (about 5–10%) of the entire sperm population is chemotactically responsive at any given time [8]. This responsive subpopulation consists of capacitated cells [8], i.e., of cells that underwent a process of maturation conferring on them, among others, the abilities to be guided to the oocyte by thermotaxis and chemotaxis [6], to bind to the oocyte, and to fertilize it [9]. It has been proposed that within a larger population of uncapacitated sperm cells residing in the oviductal isthmus, small subpopulations transiently become capacitated [8], probably ensuring a constant supply of capacitated sperm cells for fertilizing the egg over a prolonged period of time [10].

Up to now it has not been possible to isolate the chemotactically responsive cells and human sperm chemotaxis assays are usually done on a population level that comprises both responsive and (many) non-responsive cells. This makes considerable demands on the statistical handling of the experimental data.

In this report, we reconsider the earlier approach introduced by Gakamsky *et al.* [11] based on the analysis of the distribution of frame-to-frame swimming direction angles. We argue that directional persistence of sperm swimming results in correlations of successive swimming directions along a sperm track, and that this has to be taken into account appropriately by the statistical analysis of the swimming directions. We propose a method to assess the statistical significance of population measures of chemotactic performance, which is more immune to “false positives” than the original procedure. Empirical significance thresholds are determined using a variant of block bootstrapping [12] that comprises re-sampling by selection of entire sperm tracks. In our case, the “blocks” are therefore just the individual sperm tracks. We apply this method to real tracking data of human sperm cells swimming in a concentration gradient of the chemoattractant progesterone as a proof of the principle. We further illustrate this phenomenon using simulated data based on a simple mathematical model for noisy swimming paths.

## Results

### Odds ratio as a statistical measure for chemotactic performance

**Figure 1:**
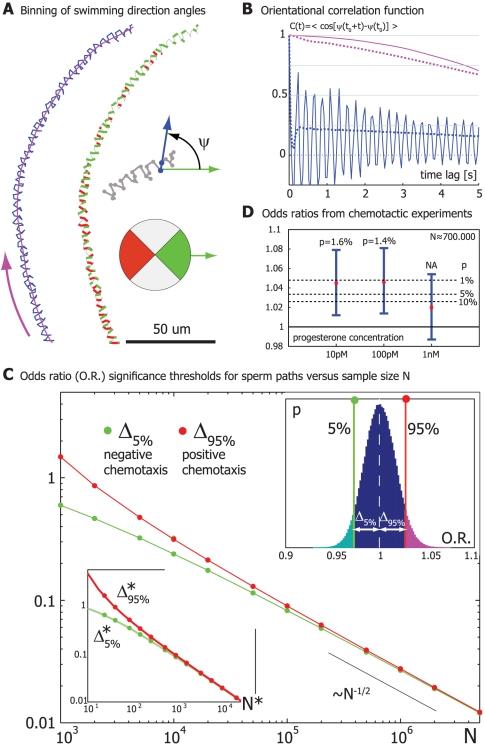
Directional persistence of sperm swimming paths prompts adapted statistical test for motion bias. **A.** One out of 30,000 control human sperm tracks (blue) and the corresponding averaged swimming path (purple; computed using a second-order Savitzky-Golay filter). The fast wiggling of the sperm head center is clearly visible. For later odds ratio calculations, angles ψ between a preferred direction and the frame-to-frame displacement vectors were binned according to the color wheel shown; the color-coded track illustrates the binning. **B.** Orientational correlation function *C*(*t*) of the swimming direction angle ψ for the sperm track from panel A (solid blue). This correlation function shows fast oscillations resulting from periodic head wiggling as well as slow decay on a time-scale of several seconds, which reflects directional persistence of sperm swimming. Also shown is a sample average of this autocorrelation (dotted blue) computed by averaging individual angle autocorrelation functions from *n* = 4,000 long sperm tracks (duration >10 sec). We can further define an analogous angle autocorrelation function for the direction angle of the averaged path (solid purple: for the averaged path from panel A; dotted purple: sample average). **C.** Empirical significance thresholds for the odds ratio of swimming direction angles for a human sperm population assay: An odds ratio O.R. = (*N*_+_/*N*_−_)/(*N*_+_^0^/*N*_−_^0^) greater than 1+Δ_95%_(*N*) with sample size *N* = min(*N*_+_+*N*_−_,*N*_+_^0^+*N*_−_^0^) should be statistically significant for positive chemotaxis at a 5%-confidence level. The test for negative chemotaxis reads O.R.<1−Δ_5%_. Significance thresholds were determined by block bootstrapping based on a large control data set of swimming direction angles of 30,000 sperm tracks. For various sample sizes *N*, we sampled a distribution of odds ratios by computing odds values for suitable random subsamples of size about *N*. Each subsample comprises the full angle data corresponding to a random selection of tracks. *Upper inset*: Distribution of odds ratios for *N* = 10^6^ by bootstrapping. The 5% and 95% percentiles of this distribution represent the significance thresholds 1−Δ_5%_ and 1+Δ_95%_, respectively. *Lower inset*: Significance thresholds Δ*_5%_ and Δ*_95%_ for a simulated control data set devoid of correlations as a function of test sample size N* (continuous lines, green Δ*_5%_, red Δ*_95%_). We obtain almost identical “significance thresholds”, if we employ simple bootstrapping drawing subsamples from *pooled* experimental angle data (not shown). The significance thresholds determined by block bootstrapping (open symbols, green Δ_5%_, red Δ_95%_) superpose with those for the simulated control data if we renormalize sample size as N* = 0.029*N*, *i.e.* Δ*_5%_(N*) ≈Δ_5%_(*N*) and Δ*_95%_(N*)≈Δ_95%_(*N*). N* can be regarded as an effective number of independent data points in an experimental sample of size *N*. **D.** Odds ratios characterizing biased motion of human sperm cells in a concentration gradient of the chemoattractant progesterone for various initial concentrations (black dots). Errorbars denote symmetric 90%-confidence intervals that were determined using bootstrapping based on the data from this particular experiment. Using bootstrapping on a separate, very large control data set, we can assign accurate significance levels *p* to each odds ratio. These significance levels represent the likelihood that the odds ratios in this particular experiment were drawn from the control distribution.

In experiments, sperm cells swim in a plane, close to a boundary surface of the observation chamber [13], [14]. Each recorded planar sperm trajectory is characterized by a time-series of swimming directions of the frame-to-frame displacement vectors. The pool of all these swimming directions from many sperm cells represents a convenient data set that is robust with respect to errors of the tracking algorithm, which can for example occur if paths cross [11]. Even if it is not known which of the observed cells are indeed chemotactically responsive, chemotactic behavior of a subpopulation of cells shows up in this population data set as a non-uniform distribution of swimming direction angles. The odds value *N*_+_/*N*_−_ is a common measure for the non-uniformity of a large data set of angles and is defined as the ratio of the number *N*_+_ of angles pointing up-gradient (−45°<ψ<45°) divided by the corresponding number *N*_−_ of angles pointing down-gradient (135°<ψ<225°), where angles ψ are measured relative to the gradient direction (inset in Figure 1A). This odds value *N*_+_/*N*_−_ has to be compared to the odds value *N*_+_^0^/*N*_−_^0^ obtained for no-gradient control conditions and the odds ratio is defined as
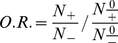
(1)Generally, values of the odds ratio larger than unity characterize positive chemotaxis towards a chemoattractant, whereas values smaller than unity characterize negative chemotaxis away from a chemorepellent. A question arises, however, concerning appropriate significance thresholds to interpret experimentally determined odds ratios. We emphasize that the standard formula for the confidence interval of an odds ratio [15] should not be applied: This formula assumes statistical independence of data points. We will see below that successive angles along the same paths are correlated.

### Directional persistence of sperm swimming paths

Figure 1A shows the planar trajectory of the sperm head of a human sperm cell swimming in a shallow observation chamber. This trajectory reveals a fast wiggling motion of the sperm head with the frequency of the flagellar beat that results from counterbalancing forces from the beating flagellum [16]. We can define an averaged swimming path that averages over several beat cycles, see the purple curve. This averaged swimming path reveals directional persistence of sperm swimming. This directional persistence of sperm swimming is also visible in the orientational correlation function [17] for the swimming angle
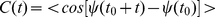
(2)This orientational correlation function shows fast oscillations (reflecting sperm head wiggling), and attains elevated values even after several seconds (reflecting continued directional persistence, Figure 1B). Of note, this correlation function cannot be described by a simple exponentially decaying function as in the case of the simple persistent random walk model, see eq. (4) below. Instead, the decay of this orientational correlation function is characterized by more than one time-scale, which include a fast time-scale of rapid head wiggling as well as a slow time-scale at which the net swimming direction changes.

### Significance thresholds for experimental odds ratios by bootstrapping

Gakamsky *et al.* [11] used bootstrapping on control data of frame-to-frame direction angles to empirically determine significance thresholds as a function of sample size for a population measure of chemotactic performance (in their study, a suitable χ^2^-value). This method of bootstrapping is reviewed in [18]: multiple selection of sub-data sets from a large control data set mimics the repetition of control experiments and allows one to accurately assess the expected variability of a quantitative measure like the odds ratio or χ^2^-value. Special care has to be taken, however, if successive data points are correlated. In our particular case, angle data from different sperm tracks can be considered independent, whereas successive swimming direction angles from the same sperm track are correlated (see above).

Due to these angle data correlations, simple bootstrapping that constructs subsamples as random selections from pooled angle control data can be problematic. To avoid these problems, we use a variant of block bootstrapping: For a given test sample size *N*, our algorithm constructs subsamples comprising approximately *N* angles that consist of all the angles corresponding to a random selection of tracks (selection with replacement, details in section ‘Materials and Methods’). Odds ratios are then computed for random pairs of sub-samples. Multiple repetition of this procedure yields a unimodal control distribution of odds ratios (upper inset in Figure 1C). The median of this distribution is equal to unity within experimental error. This distribution reflects the variability of the odds ratio that is expected due to small sample number. From this distribution we can read off the 95% percentile 1+Δ_95%_. By definition, exactly 5% of the control odds ratios will be *above* the 95% percentile (upper inset in Figure 1C, upper tail of histogram colored magenta). We will use the 95% percentile as an empirical significance threshold to test for positive chemotaxis (see section ‘Chemotactic experiments’). The rate of “false-positives” (type-I error) for this upper-tail test is 5%: Under the assumption that the null hypothesis holds true (no chemotaxis), the likelihood for observing in an experiment a particular odds ratio that is larger than the 95% percentile of the control distribution is by definition 5%. Thus, by design, this significance threshold corresponds to a significance level of 5%. Similarly, we can employ the 5% percentile, 1− Δ_5%_, as a significance threshold to test for *negative* chemotaxis. Exactly 5% of the control odds ratios will be *below* the 5% percentile (upper inset in Figure 1C, lower tail of histogram colored cyan).

Note that for small *N*, the 5% and the 95% percentiles are not symmetric, reflecting the skewness of the odds ratio distribution. For large *N*, the difference between percentiles and the median scales like the inverse square root of *N*, consistent with the law of large numbers (Figure 1C).

Under the assumption that directional persistence of sperm swimming is not significantly enhanced in the presence of a potential chemoattractant, a significant O.R. is thus a strong and robust indicator of positive taxis. To verify this assumption in our chemotactic experiments, we compared the averaged orientational correlation function for the case of sperm swimming in a concentration gradient of Progesterone (1 nM) with that for control conditions and found very good agreement (not shown). We emphasize that the block construction of sub-samples during bootstrapping is pivotal to account for the correlations that are hidden in the data and to predict correct significance thresholds.

### Consistency check of test design

We checked our test protocol for consistency of test design by determining the frequency of false-positives (“type-I-errors”). We first computed odds ratios for 28 disjoint pairs of control data sets not used before. (Each individual control data set comprised a total of N≈50,000 angles in the “up-gradient” and the “down-gradient” bin together.)

We compared these control odds ratios against the significance threshold as computed by block bootstrapping in the preceding section (1+Δ_95%_ = 1.30 for N = 50,000). One out of the 28 odds ratios exceeded this threshold. This absolute frequency of false-positives amounts to a relative frequency of 3.6% (with a symmetric 95%-confidence interval for the relative frequency equal to [0.2%, 20.2%] according to references [14], [19]). This relative frequency of false-positives is close to the chosen significance level of 5% indicating a consistent test design.

We also applied a test protocol published previously in [11] to the same 28 pairs of control data and found a rate of false-positives (12 out of 28, 43%, confidence interval [25.0%, 62.6%]) that was significantly higher than the anticipated significance level (5%). This inconsistency could be traced back to the use of simple bootstrapping instead of block bootstrapping in this former study (see Text S1). Nevertheless, published results are still statistically significant even if the new, more stringent significance thresholds are used [20].

### Chemotactic experiments

As a proof of the principle for our new statistical method, we present a particular chemotactic experiment of human sperm cells swimming in a concentration gradient of the chemoattractant progesterone (Figure 1D). Progesterone at picomolar concentrations has been previously demonstrated to be a chemoattractant for human sperm *in vitro* [21]. There is furthermore evidence for a chemotactic role of progesterone also in the physiological context, possibly guiding sperm cells to the cumulus cells surrounding the oocyte [20]. Here, we reconfirm sperm chemotaxis *in vitro* in response to progesterone at picomolar concentrations using our improved statistical method. By tracking more than *n* = 9,000 sperm cells for each experimental condition, we were able to obtain statistically significant odds ratios for applied progesterone concentrations of 10 pM and 100 pM despite the fact that the chemotactic bias was low on the population level and, in particular, not detectable by eye. Significance thresholds were determined by block bootstrapping based on an extensive control data set comprising *n* = 30,000 sperm tracks. We remark that a large set of tracking data is essential to achieve statistical significance.

For a nanomolar concentration of progesterone, the obtained odds ratio was not statistically significant. This is consistent with earlier findings that showed reduced chemotaxis at nanomolar progesterone concentrations [21]. Quite generally, it is to be expected that the dependence of a chemotactic response on the chemoattractant concentration is not a saturation curve but rather peak-like, because saturating chemoattractant concentrations can impede the detection of a concentration gradient [6].

### A simple mathematical model of stochastic swimming paths with tunable directional persistence

**Figure 2:**
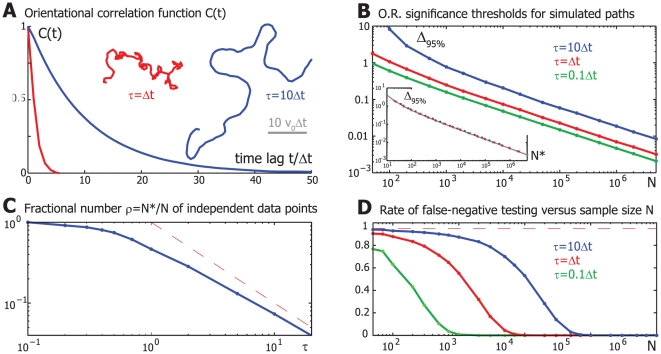
Effect of tunable directional persistence on tests of motion bias for a simple mathematical model of stochastic swimming paths. **A.** Orientational correlation function *C*(*t*) of the (frame-to-frame) swimming direction angle of *simulated* persistent random walks for two values of the persistence time τ = Δ*t* (red) and τ = 10Δ*t* (blue). Also shown are two example tracks. **B.** 95% percentile of the odds ratio distribution of simulated persistent random walks as a function of sample size *N* for different values of the persistence time τ (green: τ = 0.1Δ*t*, red: τ = Δ*t*, blue: τ = 10Δ*t*). (For each value of τ, we used simulated control data sets each comprising 10^4^ persistent random walks of duration 100Δ*t*.) *Lower inset*: Significance threshold Δ*_95%_ for a simulated control data set devoid of correlations as a function of test sample size N* (magenta line). The significance thresholds determined by block bootstrapping of simulated persistent random walks superpose with those for the correlation-free control data if we renormalize sample size as N* = ρ*N*, *i.e.* Δ*_95%_(N*) ≈Δ_95%_(*N*). N* can be regarded as an effective number of independent data points in a sample of size *N*. **C.** The “effective number of independent data points” N* in a sample of size *N* of angles of simulated persistent random walks is a function of the persistence time τ. The ratio N*/*N* was determined from a superposition of percentiles-curves of the odds ratio distribution for different values of τ by rescaling *N*→N* (“data collapse”), see inset of Figure 2B. **D.** We can test *simulated* biased persistent random walks for the presence of biased motion by comparing the corresponding odds ratio to “empirical” significance thresholds as computed in Figure 2B. This involves the risk of type-II errors (“false negatives”), in which biased motion is falsely classified as unbiased (the null hypothesis is falsely accepted). The rate of such a type-II errors decreases with sample size *N*, but increases with the persistence time τ of the biased persistent random walks (green: τ = 0.1Δ*t*, red: τ = Δ*t*, blue: *τ* = 10Δ*t*). Parameter: β = 0.1. (Note that curves do not superpose as a function of the effective number N* of independent data points as the mean of the odds ratio distribution <O.R.> depends on τ. This is a result of our use of frame-to-frame displacements vectors and an effective averaging of stochastic turning for τ<Δ*t*. If the hypothetical experimentalist could sample *instantaneous* swimming directions at discrete times, the corresponding <O.R.> would be largely independent of τ and the corresponding error rate curves would superpose.)

As shown above, directional persistence of sperm tracks results in correlations of subsequent swimming direction angles and thereby reduces the effective number of independent angles. To understand this phenomenon better, we now study a simple mathematical model for swimming paths, which allows us to tune directional persistence as a control parameter (example paths in Figure 2A). We will see that an increase in directional persistence reduces the effective number of independent data points (Figure 2C) and thus results in higher significance thresholds for tests of biased motion (Figure 2B).

A simple mathematical model for animal swimming paths is provided by the persistent random walk [22], [23] (inset in Figure 2A). Though not sophisticated enough to capture the complexity of sperm swimming paths (Figure 1A), this model allows us to illustrate how directional persistence affects testing of biased motion. A persistent random walk is characterized by a swimming direction angle ψ(*t*) that undergoes rotational diffusion with some rotational diffusion coefficient *D*_rot_ (that has units of an inverse time). Mathematically, the motion of a persistent random walker in two-dimensional space is given by
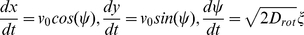
(3)where *v*_0_ is the (constant) swimming speed and ξ(*t*) denotes Gaussian white noise with variance 1. Planar persistent random walks are characterized by a decay of directional persistence on a characteristic time-scale set by the persistence time τ = 1/*D*_rot_

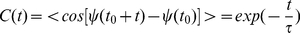
(4)In the limit of long observation times t»τ, we recover a simple random walk with effective diffusion constant *D*_eff_ = *v*_0_^2^ τ.

We now assume that the persistent random walker has the ability to align its path with respect to a uniform external field parallel to the positive *x*-axis at a rate β/τ such that its swimming direction angle now obeys
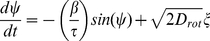
(5)Using the Fokker-Planck formalism, one shows that the angles follow a distribution that is independent of the persistence time τ, *P*(ψ)∼exp[ß cos(ψ)]. The motion of the persistent random walker is biased with a net drift in *x*-direction <Δ*x*> = *v*_0_ Δ*t* I_1_(ß)/I_0_(ß) where I_0_ and I_1_ are modified Bessel functions of the first kind.

Suppose a hypothetical experimentalist observes a number *n* of these biased persistent random walks of duration *T* each and samples each track at constant time intervals Δ*t*. As a control, *n* unbiased persistent random walks are also sampled. This experimentalist now wishes to decide about a possible bias of motion and therefore computes an odds ratio based on a bin count of frame-to-frame direction angles as described above, see eq. (1). To assess statistical significance of an observed odds value, significance thresholds are required. These can be derived by block bootstrapping as described above for a large simulated control data set of *unbiased* persistent random walks. Figure 2B shows significance thresholds 1+Δ_95%_(*N*;τ) as a function of sample size *N* = *N_+_*+*N*_−_ and correlation time τ. Interestingly, all curves superpose with a single master curve, Δ_95%_(*N*;τ) = Δ*_95%_(N*), when renormalizing sample size *N*→N*, see inset. N* can be regarded as an effective number of independent data points in an experimental sample of size *N*. The renormalization factor N*/*N* is close to one for τ<Δ*t* (low correlations between successive angles), and approximately scales as Δ*t*/τ for larger τ with Δ*t*<τ<*T*, (clusters of correlated angles have size ∼τ/Δ*t*) (Figure 2C).

Testing simulated odds values against these significance thresholds by definition correctly reproduces a rate of type-I-errors (“false positives”) equal to the significance level of 5%. The rate of type-II-errors (“false negatives”) decreases with sample size N, but increases with the persistence time τ/Δ*t* (Figure 2D).

## Discussion

In this report, we presented a test for biased motion on the population level. We compute significance thresholds for a measure of biased motion, the odds ratio, by using block bootstrapping based on an extensive control data set. The key point of our work and difference to the earlier approach [11] is to implement the bootstrapping in a way that accounts for correlations in the control data set. This is achieved by constructing sub-samples during bootstrapping that comprise a random selection of entire tracks (with “tracks” representing the “blocks”). Correlation between different tracks could result from hydrodynamic interactions [24], but can be safely ignored at the sperm densities used here. Our method allows a robust determination of significance thresholds. Testing against no-gradient controls results in a rate of false-positives that is consistent with the chosen significance level (5%). We applied this method to a population assay of human sperm chemotaxis. The specific values for the significance thresholds given here may depend on our specific experimental setup and should be computed again upon major changes of conditions. Our method is based on the odds ratio as a quantitative measure of chemotactic performance, but can easily be adapted for other measures such as a suitable χ^2^-value as used in [11].

Block bootstrapping is an established technique in the quantitative disciplines to empirically determine robust statistical measures of dependent data [12]. It has been used, for example, in the statistical analysis of particle image velocimetry data at high sampling rate [25]. In the biological sciences, however, the awareness of weak correlations in time series data, and the resulting problems for statistical analysis might be low despite existing treatments (see, e.g., reference [26]). Here, we analyzed directional swimming responses of sperm cells and thus highlighted subtleties and possible pitfalls of statistical testing of correlated biological data.

## Materials and Methods

### Spermatozoa

Human semen samples were obtained from ten healthy donors after 3 days of sexual abstinence. Informed consent was obtained in written form from each donor. Ethics approval was granted in written form by the Bioethics and Embryonic Stem Cell Research Oversight Committee of the Weizmann Institute of Science. Semen samples with normal sperm density, motility and morphology [according to WHO guidelines, [27]] were allowed to liquefy for 30–60 min at room temperature. Human spermatozoa were separated from the seminal plasma by the migration–sedimentation technique [28] using commercially available Modified HTF medium (Irvine Scientific, Santa Ana, CA, USA) supplemented with 0.3% human serum albumin (HSA, Irvine Scientific, Santa Ana, CA, USA). Following this procedure, the sperm concentration was adjusted to 4×10^5^ cells/ml in HTF medium containing 0.3% HSA and 3.5% polyvinylpyrrolidone (PVP 25K, Fluka, Buchs, Switzerland). The sperm suspensions were incubated under an atmosphere of 5% CO_2_ at 37°C for an additional 1 h (in total, 2 h together with the separation procedure) to obtain capacitated spermatozoa [8].

### Chemotaxis assay

Chemotaxis assays were performed at room temperature in a disposable μ-slide chemotaxis chamber, consisting of two large volume reservoirs connected by a thin slit (Ibidi GmBH, Munich, Germany), see also [29]. In a series of initial experiments, the establishment of a stimulus concentration gradient was verified by the use of rhodamine B. Sperm suspensions and stimulus solutions were adjusted to room temperature prior to the experiment. The chamber loading was according to manufacturer instructions: Both reservoirs and the slit of the chamber were filled with spermatozoa, thus avoiding any directional bias of the initial sperm concentration field in the direction of the concentration gradient to be applied. The chemoattractant was then applied within a sperm suspension to one of the reservoirs. This avoided dilution of the cell concentration by the application of the chemoattractant. Following loading, the slides were incubated at room temperature for 20 min to allow the establishment of a concentration gradient of the stimulus. The swimming of spermatozoa in the observation area was video-recorded at 25 frames per second for a total of 4 min using a Nikon Eclipse Ti microscope at 10× magnification. Two different fields of view were interchanged every 40 s by manually moving the microscope stage in the direction perpendicular to the concentration gradient. This procedure reduced oversampling of sperm cells swimming in tight circles. Subsequently, sperm tracks were analyzed with custom-made software.

### New bootstrap protocol

By combining control data from different sperm samples, we compiled a large control data set of 30,000 sperm tracks; for each track, the corresponding set of frame-to-frame direction angles was stored. Then, again for each track, angles outside the bins of interest (i.e. those satisfying 45°<ψ<135° or 225°<ψ<315°) were discarded. To construct a subsample of size *N*, tracks were randomly chosen from this large control data set (with replacement) until the selected tracks together made up a total of about *N* angles. (The true subsample size could deviate from *N* with standard deviation ∼40 angles, which is negligible for our purposes.) Our selection of tracks represents a variant of block bootstrapping. Using two random subsamples, an odds ratio can be computed according to eq. (1). By repeated selection of (pairs of) subsamples, we obtained a distribution of odds ratios (upper inset in Figure 1C). The 95% percentile of this distribution, 1+Δ_95%_, is shown in the main panel of Figure 1C as a function of subsample size *N*. We used this 95% percentile as the significance threshold to test data from chemotactic experiments.

### Old bootstrap protocol

For the convenience of the reader we cite the bootstrapping protocol from an earlier work [11]. There, for a pair of control data (from the same sperm sample), frame-to-frame direction angles from all tracks of each data set were pooled, thus giving rise to two angle pools. Angles outside the bins of interest (i.e. those satisfying neither −45°<ψ<45° nor 135°<ψ<225°) were discarded. Then, from each angle pool, a subsample of size *N* was drawn by random selection (with replacement). Binning of angles in these two subsamples gives the numbers of angles in the “up-gradient” bin (−45°<ψ<45°) for the two subsamples, *N1*_+_ and *N2*_+_, respectively. By construction, the number of angles in the “down-gradient” bin (135°<ψ<225°), are *N1*_−_ = *N*−*N1*_+_ and *N2*_−_ = *N*−*N2*_+_ for the two subsamples, respectively. The bin counts *N1*_+_, *N1*_−_, *N2*_+_, and *N2*_−_ represent a contingency table, from which a χ^2^-value can be computed using the standard formula χ^2^ = [(*N1*_+_−*N2*_+_)^2^/(*N1*_+_+*N2*_+_)+(*N1_−_*−*N2*_−_)^2^/(*N1*_−_+*N2*_−_)]/2. By repeated selection of pairs of subsamples, one obtains a distribution of χ^2^-values, from which a 95% percentile can be read off. Interestingly, applying this bootstrapping protocol to different pairs of control data gave quite different χ^2^-distributions, and thus a set of different values for the respective 95% percentiles. In [11], it was proposed to use the median of this set of 95% percentiles as significance threshold to test data from chemotactic experiments.

## Supporting Information

Text S1 **Supporting Information Text contains a detailed comparison of a previously published statistical test for biased motion** [**11**] **and the new test proposed here together with an analysis for the assoicated rate of false-positive-testing.**
(DOCX)
