# Supplementary material for: Testing Human Sperm Chemotaxis: How to Detect Biased Motion in Population Assays
Source: PLoS One. 2012 Mar 8;7(3):e32909. doi: 10.1371/journal.pone.0032909 (PMC3297605; doi:10.1371/journal.pone.0032909)
Supplement: Text S1 — Supporting Information Text contains a detailed comparison of a previously published statistical test for biased motion [11] and the new test proposed here together with an analysis for the assoicated rate of false-positive-testing. (DOCX) [file pone.0032909.s001.docx]

**Supporting Information**

**Testing human sperm chemotaxis:**

**How to detect biased motion in population assays**

**Leah Armon^1^, S Roy Caplan^1^, Michael Eisenbach^1^, Benjamin M Friedrich^2,3,*^**

**1** Department of Biological Chemistry, Weizmann Institute of Science, Rehovot, Israel

**2** Department of Materials and Interfaces, Weizmann Institute of Science, Rehovot, Israel

**3** Max Planck Institute for the Physics of Complex Systems, Dresden, Germany

* Corresponding author: [ben@pks.mpg.de](mailto:ben@pks.mpg.de)

**S1 Comparison of bootstrapping protocols**

The previous study from Gakamsky *et al.* proposed a bootstrapping protocol that differs from the new one proposed here in three respects (see also section ‘Materials and Methods’ in the main text):

(1) Whereas our new protocol uses block bootstrapping with track selection to account for correlations among angles from the same track, Gakamsky *et al.* employs simple bootstrapping drawing subsamples from pooled angle control data.

(2) In Gakamsky *et al.*, bootstrapping was performed not on a single, combined control data set, but instead on pairs of (smaller) control data sets. A set of 95% percentiles was thus obtained and the median of this set was proposed as significance threshold.

(3) Finally, our approach is based on the odds ratio as measure for biased motion, while Gakamsky *et al.* used a suitable χ^2^-value.

We checked both protocols for consistency of test design by determining the frequency of false-positives (“type-I-errors”).

We first computed odds ratios for 28 disjoint pairs of control data sets not used before. (Each individual control data set comprised a total of *N*≈50,000 angles in the “up-gradient” and the “down-gradient” bin together.) We compared these control odds ratios against the significance threshold as computed by block bootstrapping in the preceding section (1+Δ_95%_=1.30 for *N*=50,000). One out of the 28 odds ratios exceeded this threshold, which amounts to a relative frequency of false-positives of 3.6%. This relative frequency of false-positives is close to the chosen significance level of 5% indicating a consistent test design.

We then used the bootstrapping protocol of Gakamsky *et al.* to compute a tentative significance threshold for χ^2^-values from nine pairs of control data sets (with *N*≈40,000-100,000). For subsample size *N*=50,000, we found as threshold χ^2^_thresh_=16.7 (median of a distribution of nine 95% percentiles with minimum 3.9 and maximum 47.8). We compared the χ^2^-values from the same 28 pairs of control data sets used before against this threshold and obtained 12 false-positives, which amounts to a relative frequency of 43%. Even if we had used as threshold the maximum of the ten 95% percentiles, there would have still been 7 false-positives. If the true rate of false-positives were indeed 5%, the likelihood for observing 7 or more false-positives out of 28 tests is <0.4%. We found similar results by applying this test to the very same control data used already to bootstrap the significance thresholds. It thus seems that the approach by Gakamsky *et al.* classifies too many control experiments as statistically significant for positive chemotaxis, inconsistent with the chosen level of significance (5%).

We asked ourselves which of the three differences between the old and the new bootstrapping protocols was responsible for this inconsistency of the old protocol.

Using a variant of the new protocol for χ^2^-values gave no false-positives at all. Hence, the use of a different measure of biased motion (difference no. 3) cannot explain the difference in test consistency between the old and the new protocols.

Next, we ran simple bootstrapping for χ^2^-values as used by Gakamsky *et al.* not on pairs of smaller control data sets, but on a single, combined control data set. The tentative significance threshold computed this way (χ^2^_thresh_=3.84) was even smaller than before. Out of the 28 control odds ratios, 17 exceeded this threshold. The inconsistency of the old bootstrapping protocol can thus not be explained by the fact that several pairs of small control data sets instead of a combined control data set had been used (difference no. 2).

Hence, it seems that the crucial difference between both protocols is whether simple bootstrapping or block bootstrapping is employed (difference no. 1).

We implemented the new protocol with simple bootstrapping instead of block bootstrapping, and obtained tentative significance thresholds (lower inset of Figure 1C) that are much smaller than those predicted using block bootstrapping (Figure 1C main graph). For example, for sample size *N*=50.000, we obtain 1+Δ_95%_=1.130 by block bootstrapping, but the much lower value 1+Δ_95%_*=1.021 using simple bootstrapping. If we compare the 28 control odds ratios against this lower (incorrect) threshold, we obtain 6 false-positives, which amounts to a relative frequency of 21%. Testing against an analogous, tentative significance threshold for negative chemotaxis 1-Δ_5%_*=0.979 resulted in 11 out of 28 (=39%) false-positives.

*Technical remark:* Mathematically, the selection (with replacement) of a subsample from an angle pool in the old bootstrap protocol followed by the determination of the number *N*_+_ of angles in the “up-gradient” bin is fully equivalent to a Binomial trial consisting of *N* Bernoulli experiments with probability *p* that counts the number *N*_+_ of positive outcomes. Here *p* is the relative fraction of angles in the “up-gradient” bin in the angle pool. This equivalence allows speeding up the corresponding algorithm considerably. This equivalence also helps to appreciate the apparent variability in the distribution of χ^2^-values when the old bootstrap protocol is applied to different pairs of control data. The respective relative frequencies of angles in the “up-gradient” bin for the two angle pools, p1 and p2, should have the same expectation value since they were derived for identical experimental conditions. If, indeed, p1 and p2 were equal, the bootstrap protocol would always report a 95% percentile equal to 3.84 - the regular critical χ^2^-value for χ^2^-tests with one degree of freedom. However, due to the finite size of the control data sets, p1 and p2 will, in general, slightly deviate from each other. This results in a dependence of the 95% percentiles on the subsample size *N* as reported in Gakamsky *et al*. This dependence is stronger the more p1 and p2 deviate from each other and thus varies for different pairs of control data.

**References**

Gakamsky A, Schechtman E, Caplan SR, Eisenbach M (2008) Analysis of chemotaxis when the fraction of responsive cells is small - application to mammalian sperm guidance. Int J Dev Biol 52: 481-487.
